# Editorial for the Special Issue: “Tick-Borne Encephalitis”

**DOI:** 10.3390/microorganisms11040934

**Published:** 2023-04-03

**Authors:** Daniel Ruzek

**Affiliations:** 1 Faculty of Science, Masaryk University, CZ-62500 Brno, Czech Republic; ruzekd@paru.cas.cz; 2 Institute of Parasitology, Biology Centre of the Czech Academy of Sciences, CZ-37005 Ceske Budejovice, Czech Republic; 3 Laboratory of Emerging Viral Infections, Veterinary Research Institute, CZ-62100 Brno, Czech Republic; 4 Research Center for Thermotolerant Microbial Resources, Joint Faculty of Veterinary Medicine, Yamaguchi University, Yamaguchi 753-0841, Japan

Tick-borne encephalitis (TBE) is a disease caused by the tick-borne encephalitis virus (TBEV). It may be asymptomatic or often limited to a febrile illness, but can also lead to very aggressive neurological sequelae [1]. The disease is widespread in forested areas of Europe and northeast Asia [2]. Humans are usually infected through the bite of a TBEV-infected tick, but alimentary infection after consumption of unpasteurized milk or milk products from infected ruminants is also possible [3]. The number of TBEV cases in humans has increased dramatically in recent decades in some of the endemic regions, particularly in northern Europe; risk areas have expanded and new foci have been discovered [1]. The only effective protection against TBE is active vaccination. Currently, there is no specific antiviral therapy to treat TBE [4].

The Special Issue, “Tick-borne Encephalitis”, in *Microorganisms* contains the latest contributions to the field of TBE research. A total of 11 research papers and one case report were published in this Special Issue. The published papers focus primarily on the clinical aspects, pathogenesis, ecology and epidemiology of TBE.

The article by Kohlmaier et al. [5] summarizes the clinical data and laboratory characteristics of a large international cohort of TBE patients. A total of 555 patients were recruited between 2010 and 2017 in 8 centers from 6 European countries. The study observed high rates of patients with encephalitis and high rates of patients with persistent signs and symptoms at discharge. Interestingly, the cohort also included 16 patients with a history of TBE vaccination, and among these there was a high proportion of children and adolescents, as 9 of the vaccine breakthroughs were reported in patients younger than 20. Overall, this is the first international multicenter study of TBE patients from different European countries, providing an impressive collection of clinical data and improving our understanding of the clinical course and presentation of TBE.

Typically, TBE has two phases, with the first phase characterized by nonspecific symptoms such as fever, headache, muscle pain and fatigue, followed by the second neurological phase after a few days of improvement. A monophasic course is also possible, although there is very little information on such cases. The study by Bogovic et al. [6] examined clinical presentation, laboratory findings and immune responses in a large cohort of TBE patients that included 283 cases with a monophasic course and 422 cases with a biphasic course. Compared with the patients with a biphasic course, the patients with a monophasic course were older, more likely to have comorbidities and more frequently admitted to the intensive care unit, suggesting more severe disease. This underscores the importance and potential severity of monophasic TBE cases and also has practical implications for patients and their physicians, who should be aware of this atypical disease presentation, which may be associated with a higher risk of a severe course.

In another study by Bogovic et al. [7], disease severity was also found to be associated with a low serum TBEV-specific IgG antibody response, as shown by analysis of serologic responses in a large cohort of TBE patients comprising 691 individuals. The study found that low serum IgG levels at the time of onset of the neurologic phase of the disease correlate with a more severe course and unfavorable long-term outcome of TBE. Therefore, low TBEV-specific IgG levels detected at the onset of the neurologic phase could serve as a predictor for a higher risk of more severe disease and the development of post-encephalitic syndrome.

TBE infections in children are usually thought to be milder than in adult patients, although severe cases can occur. One such case is described in a case report by Krbkova et al. [8], who reported on severe TBE in an 8.5-month-old boy who had severe seizures at the onset of the neurologic phase. To date, very few reports of infants with TBE in the first year of life have been published, and this article provides important information about the potential severity of TBEV infection in children. It also contributes to recent discussions about whether TBE vaccination should be extended to children younger than 1 year of age living in areas with high TBE incidence.

The importance of TBE vaccination is also highlighted in the article by Chitimia-Dobler et al. [9], which provides evidence that TBE vaccination protects not only against TBEV infection from ticks, but also against alimentary TBEV infection. The article reports an outbreak of alimentary TBE infection after consumption of raw milk from a goat farm in Germany. None of the exposed individuals who were vaccinated against TBE developed clinically manifest TBE, whereas 12 of 14 unvaccinated and exposed individuals had clinical TBE, demonstrating that the alimentary TBEV infection can be effectively prevented by vaccination.

Gastrointestinal symptoms occur sporadically in human TBE cases. Boelke et al. [10] used a TBE mouse model to examine gastrointestinal symptoms in more detail to understand the mechanisms behind this pathologic manifestation. They found that ganglioneuritis is a common feature in TBEV-infected mice, suggesting immune-mediated pathogenesis due to TBEV infection of the enteric plexus. TBEV infection may therefore be associated with damage to neurons of the enteric nervous system, which could be a cause of the acute and persistent gastrointestinal pathology and dysmotility observed in TBE.

TBE cases may still be underreported in some endemic areas, as shown in a study by Yoshii et al. [11]. Their surveillance study revealed undiagnosed TBE in patients with neurologic disorders and previously unreported cases of TBEV infection in an endemic region in Hokkaido, Japan, highlighting the importance of further surveillance studies in this region and also the need to consider the establishment of a TBE vaccination program to control TBE in Japan.

Walter et al. [12] developed a dataset of georeferenced TBEV detections in ticks and endothermic mammals that was used in conjunction with environmental variables to map the probability of TBEV occurrence in different parts of Europe. This approach may identify new areas associated with the risk of TBEV infection in humans. The study by Liebig et al. [13] showed that certain TBEV isolates might be associated with certain tick populations and that this virus–tick relationship might be responsible for the focal distribution of TBEV in nature. Deviatkin et al. [14] also provided new information on the role of flying animals in the spread of certain TBEV strains and their mixing between distant locations.

Various wild or domestic animals can be used as sentinels for TBE. Haut et al. [15] collected serum samples from 1233 red foxes (*Vulpes vulpes*) in Germany and examined them for the presence of TBEV-specific antibodies via an ELISA and indirect immunofluorescence assay (IIFA). The results were also confirmed by a micro-neutralization test (micro-NT). They found that seropositivity rates differed significantly between TBEV risk and non-risk areas, suggesting that red foxes are a promising surrogate marker for TBE. The importance of the micro-NT in TBE serosurveys was further emphasized in a study by Girl et al. [16], who evaluated the use of dogs as sentinel animals in southern Germany. They found that the neutralization test remains the gold standard for the detection of TBEV-specific antibodies, which cannot be fully replaced by ELISA or IIFA.

In summary, the collection of articles published in this Special Issue of *Microorganisms* highlights some important advances in TBE research. It addresses the topic from a broad perspective and in a comprehensive way, and includes studies of TBE in humans, the pathogenesis of TBE using animal models and, last but not least, studies of the ecology and epidemiology of TBE. The articles also advert to gaps in the knowledge and raise new questions to be addressed in future studies to gain a more comprehensive understanding of this important disease.
